# Predictive modeling in biology and medicine: Digital twins and multi-scale modeling

**DOI:** 10.1371/journal.pcbi.1014349

**Published:** 2026-06-04

**Authors:** Mark Alber, Amber Smith, Reinhard Laubenbacher, Roeland M. H. Merks

**Affiliations:** 1 Department‌‌ of Mathematics, University of California, Riverside, Riverside, California, United States of America; 2 Interdisciplinary Center for Data-Driven Modeling in Biology, University of California, Riverside, Riverside, California, United States of America; 3 Department of Pediatrics, University of Tennessee Health Science Center, Memphis, Tennessee, United States of America; 4 Department of Microbiology, Immunology, and Biochemistry, University of Tennessee Health Science Center, Memphis, Tennessee, United States of America; 5 Institute for the Study of Host-Pathogen System, University of Tennessee Health Science Center, Memphis, Tennessee, United States of America; 6 Department of Medicine, University of Florida, Gainesville, Florida, United States of America; 7 Mathematical Institute and Institute of Biology, Leiden University, Leiden, The Netherlands; Johns Hopkins University, UNITED STATES OF AMERICA

## Digital twins

Over‌‌ the last 10 years, it has been shown that multi-scale modeling approaches combined with machine learning provide a powerful method for developing robust predictive models for studying many biomedical processes and exploring massive data sets [1,2]. Recently, the concept of *digital twins* has gained increasing attention in biology, biomedicine, and healthcare communities. This collection explores the construction of digital twins and multiscale models as they relate to predictive biology and medicine.

## Origins

The concept originated in work at NASA and is broadly used in industry and other fields, such as city planning. In the biological context, a digital twin of a biological system, whether a specific animal or patient, a specific corn field, or the population of a village, is a computational model of some aspect of the system and is calibrated dynamically to evolve together with the system, linking the physical and the digital twin. The digital twin can then be used to identify interventions or features of interest of the physical twin. This approach to “personalized” biology has tremendous potential for biotechnology, ecology, and healthcare in the future, to name just a few application areas. For a comprehensive discussion of this topic see the 2023 report on this topic by the National Academies of Science, Engineering, and Medicine (NASEM) [3] and reviews [4,5].

## Immune digital twins

Recent advances in mathematical and computational modeling are accelerating progress toward digital twins in biology and medicine, which are integrated, predictive systems that represent biological function across scales. The three studies featured in this collection illustrate complementary directions in this emerging field. Fonseca and colleagues examine the theoretical and methodological foundations for constructing digital twins, introducing optimal control frameworks that integrate mechanistic and data-driven modeling approaches. Their work addresses challenges in adapting optimal control to complex, stochastic biological systems, particularly those involving spatially structured immune responses. Complementing this, Krumpinky and colleagues. employed a multi-scale compartmental model of *Mycobacterium tuberculosis* infection to explore how granuloma formation and lymphatic trafficking jointly influence immune regulation and disease variability within and across lung and lymph node environments. Badrou and colleagues advance the structural modeling of the human lung through inverse finite element analysis, developing a continuously ventilated, anatomically representative model that integrates tissue- and organ-level experimental data. Among other important applications, model predictions of strains can help clinicians during mechanical lung ventilation.

## Predictive computational models of physiological processes with biomedical applications

Three papers develop predictive computational models for studying mechanisms of blood coagulation and for designing new types of bioartificial organ scaffold in a blood vessel. The paper by Ouedraogo and colleagues developed a 2-dimensional stochastic kinetic model of a fibrin fiber cross-section that uses the Gillespie algorithm to study plasmin-mediated degradation of fibrin. The authors indicate that experimentally calibrated model simulations predict lysis rates and the level of fiber degradation before cleavage. Obtained better understanding of the mechanisms of impaired fibrinolysis could inform patient-specific treatment of different states of thrombosis. The paper by Santiago and colleagues used biologically calibrated mathematical models and adaptive Metropolis method to determine the best representation of the biochemical reactions involving TFPI inhibition of Factor X activation by the TF-VIIa complex in blood coagulation cascade. Adding obtained results to larger models could provide novel understanding of clotting disorders and help with development of new therapies. The paper by Qiao, Getz and colleagues used 3D reaction-diffusion model with a Gaussian distribution-modeled AKAP/AC nanodomain to explore in-phase and perfectly out-of-phase Ca^2+^ and cAMP oscillations mediated by being inside and outside the nanodomains. The results of this paper demonstrate how molecular spatial positioning can regulate temporal dynamics of second messengers in different cellular locations. The authors indicate that the impact of membrane nanodomains on the local profiles of oscillations can play an important role in many diseases and developmental and physiological processes. In the paper Ulloa and colleagues, the authors use combined experiments and model simulations to study potential role of an external uniaxial force applied during puberty on the regulation of the orientation of epithelial ducts in the ductal network. Branching and annihilating random walk model simulations demonstrate that changes in the orientation of the epithelial ducts can lead to an increase in the length of the ductal network and change in duct orientation.

## New computational methodology for biomedical application

The paper by Bottcher and colleagues describes a statistical model for resolving uncertainties related to false positive and false negative diagnostic errors. It is used to improve medical decision-making by combining outcomes of multiple diagnostic and screening tests. This approach can be used for increasing test efficiency in clinical and public health contexts. The paper by Wu and colleagues develops methodology for data-driven model discovery and model selection based on hybrid dynamical systems, partial ordinary differential equations models with missing terms. The paper focuses on overcoming the limitations in learning additional terms in the equations when using sparse and noisy input data by applying neural networks and sparse and symbolic regression approaches. The methodology is successfully demonstrated using the Lotka–Volterra model from population dynamics and the repressilator, an example of a genetic regulatory network.

The paper by Jasuja and Atzberger develops a novel multi-physics theoretical and computational modeling approaches based on Stochastic Eulerian Lagrangian Methods which provide stochastic hybrid continuum-discrete descriptions of individual protein dynamics with spatially varying concentration fluctuations and thermal exchanges. The developed methodology approach has important applications for studying non-equilibrium phenomena in a variety of biological systems, active soft materials, and complex fluids. In predictive modeling it often occurs that sets of similar, or even complementary assumptions at the microscopic level may yield very similar predictions at the macroscopic level. This becomes problematic, for example, if we are using the model to predict potential intervention strategies. Thus, strategies for falsifying models are necessary. The paper by Vergroesen and colleagues compares and verifies simulations obtained using three cell-based models of cellular Potts type of endothelial cell migration and organization during angiogenesis with time-lapse videos of endothelial cell network formation in *in vitro* experiments. By varying initial cell densities in different models’ simulations and in the *in vitro* experiments, it is shown that the cell elongation model best captures the remodeling phase of the endothelial cell network formation. The approach described in the paper highlights the importance of evaluating models using time-resolved data and provides a framework for further development of biologically relevant models of angiogenesis. Conte and colleagues provide an extended analysis of the identifiability of parameters of transport nested compartment models extensively used for the analysis of DCE-MRI data for determining both structural and practical identifiability. In particular, the paper studies the impact of noise and smoothening on the identifiability. Obtained results on determining reliability and reproducibility of transport model predictions are of immediate importance due to the wide use of DCE-MRI data in biological research and the medical field.

## Examples of predictive modeling at the subcellular level

Nguyen and colleagues study the general binding process of a kinesin motor to a microtubule by modeling a simplified system of single motor attached to a bead held at a specific distance from the microtubule. The time of a kinesin motor binding to a microtubule is determined by combining experimental optical trapping with a mechanistic model with an ADP-state dependence. Tsai and colleagues describes a three-dimensional mathematical/computational multi-scale chemical-mechanical modeling framework for studying polarized growth in response to a pheromone in aging cells in yeast. Signaling reaction-diffusion sub-model in local curvilinear coordinates in the form of Laplace–Beltrami equations solved on the growing 2D membrane in 3D space is coupled with a mechanical sub-model of a growing membrane. Multi-scale model simulations suggest and test several mechanisms of morphological changes in aging yeast which can be of importance in aging of other cell types. The paper by Cannon and colleagues studies how bacteria *Rhodospirillum rubrum* dissipate chemical energy obtained by the bacteria through photosynthesis. The authors combine an extensive genome-scale bacteria metabolic model with models of mass action kinetics consistent with the thermodynamics of reactions and pathways to study “redox poise” in non-sulfur bacteria (PNSB). Based on model simulations and experimental data, the authors conclude that nucleotide and protein production could be an important mechanism of maintaining redox balance.

## Conclusions and vision

Together, the papers from this special collection demonstrate how computational methodologies ranging from control theory and multi-scale immunology and physiology to biomechanical modeling provide the necessary building blocks for the realization of translational digital twin systems capable of predicting disease progression and informing personalized interventions. The models presented in this special collection unite experimental biological and clinical data within coherent mechanistic frameworks and consider a range of biological levels of organization from the protein and subcellular scale all the way up to the physiological scale.

While the development of a digital twin involves predictive modeling, a core activity featured at the conference involved several other components. As the NASEM report describes, there needs to be a bidirectional connection between the model and the physical object being modeled. It is only this connection that makes the model into a digital twin. Thus, we need to develop methods for data assimilation that allow the dynamic updating of the model with data from the physical object. Also, we need to develop novel multi-scale methodology for coherently coupling biological levels of organization and their mutual interactions to a model of a human organ or even the whole body, such that interventions at one scale—often pharmacologically at the molecular scale—can be followed through up to its effect on the organ or whole human body. Such predictive, composite multiscale digital twins would inevitably lead to huge computational challenges. Solutions to such challenges are recently found by replacing part of the model, e.g., one of the biological levels of organization, by an AI counterpart that can deliver useful predictions much more quickly than the fully mechanistic methods that we have focused on in this special collection. Two promising avenues in this direction are currently emerging. If only the input/output relation is of interest for a particular scale, such as response of single cells in an organ scale model, the scale outside the direct scale of interest can be approximated using a statistical, machine learning model that is trained on real data or a detailed simulation (see, e.g., response of neurons in a model of peristalsis of the esophagus [6]. In other cases, we may need more “inside” information of a biological scale, while the details underlying the formation of the scale may be irrelevant. A good example is the plasticity of a blood vessel network inside the *digital twin* of an organ such as the liver. For such cases, a promising new concept is provided by surrogate modeling, which captures the plastic response of a complex, mechanistic model in a more computationally efficient AI-based model [7]. Altogether, the combination of AI-methodology and mechanistic, multi-scale modeling is a promising direction for developing computationally efficient *digital twins* while maintaining the required mechanistic detail necessary for biologically and medically meaningful predictions.

After a computationally efficient *digital twin* has been constructed, development of novel methodology is needed to use it for forecasting and the design of interventions. Because many biological systems and applications have features that do not lend themselves easily to modeling with ordinary differential equations, other types of models such as agent-based models or data-driven/AI models will need to be considered. There is no off-the-shelf suite of tools available for forecasting and control purposes. Thus, there are many opportunities for the predictive biomedical modeling community to engage in research on this topic. A particular challenge is the highly interdisciplinary nature of any such project. For instance, when it comes to applications in medicine, any *digital twin* project will need to involve clinicians, biologists, mathematical modelers, and computational scientists. While very substantial progress has been made during the last decade, integrating biology and computational modeling faces new challenges, and even more heterogeneous interdisciplinary research teams are needed for the development of *digital twins*.
